# Simultaneous infrared thermal imaging and laser speckle imaging of brain temperature and cerebral blood flow in rats

**DOI:** 10.1117/1.JBO.24.3.031014

**Published:** 2018-11-22

**Authors:** Takashi Suzuki, Naoya Oishi, Hidenao Fukuyama

**Affiliations:** a Kyoto University, Research and Educational Unit of Leaders for Integrated Medical System, Center for the Promotion of Interdisciplinary Education and Research, Kyoto, Japan; b Beijing Institute of Technology, Human Brain Research Laboratory, Intelligent Robotics Institute, Beijing, China

**Keywords:** brain temperature, cerebral blood flow, infrared imaging, laser speckle imaging, multimodal imaging, neurovascular metabolic coupling

## Abstract

Infrared thermal imaging of brain temperature changes is useful for evaluating cortical activity and disease states, such as stroke. However, the changes depend on a balance between changes in heat generation from metabolism and in heat convection related to blood flow. To discriminate between these effects and gain a clearer understanding of neurovascular metabolic coupling, brain temperature imaging must be improved to measure temperature and blood flow simultaneously. We develop an imaging technique that shows a two-dimensional (2-D) distribution of absolute brain temperature and relative cerebral blood flow changes in anesthetized rats by combining infrared thermal imaging with laser speckle imaging. The changes in brain metabolism and cerebral blood flow are achieved using two different anesthetics (isoflurane and α-chloralose) to evaluate our system. Isoflurane increased cerebral blood flow but decreased metabolism, whereas α-chloralose decreased both parameters. This technique enables simultaneous visualization of brain surface changes in temperature and cerebral blood flow in the same regions. This imaging system will permit further study of neurovascular metabolic coupling in normal and diseased brains.

## 1 Introduction

Regional surges in neuronal activity are accompanied by changes in local brain metabolism, cerebral blood flow, and cerebral oxidation. The nature of the relationship between neural activity changes, metabolic changes, and cerebral blood flow changes is commonly known as neurovascular metabolic coupling,^1^^–^^4^ the mechanism of which remains unclear. Clearer understanding of this coupling will enable greater insight into brain function and be useful in the diagnosis and treatment of neurovascular diseases, such as acute stroke accompanied by neurovascular metabolic uncoupling.^5^ In rat brains in which stimulus-induced vasodilation was suppressed using a calcium channel blocker, it has also been reported that neurovascular metabolic uncoupling, such as brain temperature increases without cerebral blood flow, changed during stimulation.^6^ Therefore, simultaneous monitoring of spatiotemporal changes in cerebral blood flow and cerebral metabolism is important.

Regional metabolic activity in the brain, which is the primary mechanism that satisfies the energetic demands of the brain,^7^^,^^8^ generates heat and increases local brain temperature concurrent with cerebral blood flow changes.^9^ Temperature changes may be more closely related to actual neural activity than cerebral blood flow changes. Thus, the measurement of absolute change in the local brain temperature may allow quantification of neural activity.

Localized thermal changes from direct measurement using thermistors have been reported in cat brain with visual and auditory stimulation.^10^ Visual stimulation raised the temperature in the lateral geniculate nucleus by 0.001°C to 0.1°C, and auditory stimulation raised the temperature in the inferior colliculus by 0.001°C to 0.0015°C. In the freely moving rat, the brain temperature increased prior to and greater than the increase in blood temperature evoked by a stimulus.^9^ Weber et al.^11^ also indicated that the increase in cerebral oxidative metabolism in response to sensory stimulation was considerably faster and more localized than the cerebral blood flow response in rats.

Thermal imaging by an infrared camera can noninvasively create a temperature map with high spatial, temporal, and temperature resolution. The technique of infrared imaging has been applied to measure functional temperature changes in humans as an intraoperative application.^12^ We previously developed a brain temperature imaging technique to quantitatively evaluate brain activity in rats^6^ and demonstrated the utility of infrared thermal imaging for quantitative functional neuroimaging. However, we found that some temperature changes occurred closely in the cerebral vessels following neural activity. Separating the effect of cerebral blood flow from neural and metabolic activities is difficult for this imaging, and the interaction between temperature and blood flow remains ambiguous. To solve this problem, we need to measure the brain temperature and cerebral blood flow simultaneously. The use of optical imaging techniques that map hemodynamic properties has become prevalent in neuroimaging. Especially, laser speckle imaging is often used as a supplementary method for quantifying blood flow.^13^^–^^15^ Furthermore, optical imaging can expand capabilities by combining some modalities relatively easily.^11^^,^^16^^–^^19^ The option of optical techniques to evaluate both brain temperature and cerebral blood flow simultaneously can provide further information on their coupling and uncoupling.

Some simultaneous optical imaging techniques to measure cerebral blood flow and cerebral metabolic changes in rodent brains have been developed and include combining optical intrinsic signal imaging,^16^ flavoprotein autofluorescence,^11^ multispectral reflectance imaging,^17^ and phosphorescence lifetime imaging^18^ with laser speckle contrast imaging. These multimodal systems need one or more excitation light sources separate from a laser used for laser speckle imaging. Accordingly, it is necessary to design the optical system, such as by switching the light sources. In addition, there are multimodal systems that use Doppler optical coherence tomography to measure cerebral blood flow and confocal microscopy to measure cerebral oxygen partial pressure using the phosphorescence-quenching method.^19^ In this system, a more complicated optical system and techniques were required. Oxygen-dependent quenching of phosphorescence is a useful and essentially noninvasive optical method for measuring oxygen *in vivo*.^20^ However, the phosphorescence-quenching method needs administration of an oxygen-sensitive phosphorescence probe (Oxyphor R2) for the oxygen partial pressure measurement and is limited to point measurements in the multimodal imaging. To obtain a multimodal system, we developed an optical imaging system for mapping the distribution of brain temperature and cerebral blood flow that has high spatial, temporal, and temperature resolution by combining brain temperature imaging and laser speckle imaging.

We tested whether our system could detect vascular–metabolic coupling and uncoupling using two kinds of anesthesia: isoflurane and α-chloralose. Isoflurane leads to increasing cerebral blood flow^21^^,^^22^ and decreasing brain temperature.^23^ On the other hand, α-chloralose also leads to decreasing both the cerebral blood flow^24^ and the temperature.^25^ The purpose of this study was to demonstrate the potential of an optical imaging system that can simultaneously acquire temperature and blood flow changes on the cortical surface of anesthetized rats.

## 2 Materials and Methods

### 2.1 Animal Preparation

**Fig. 1:**
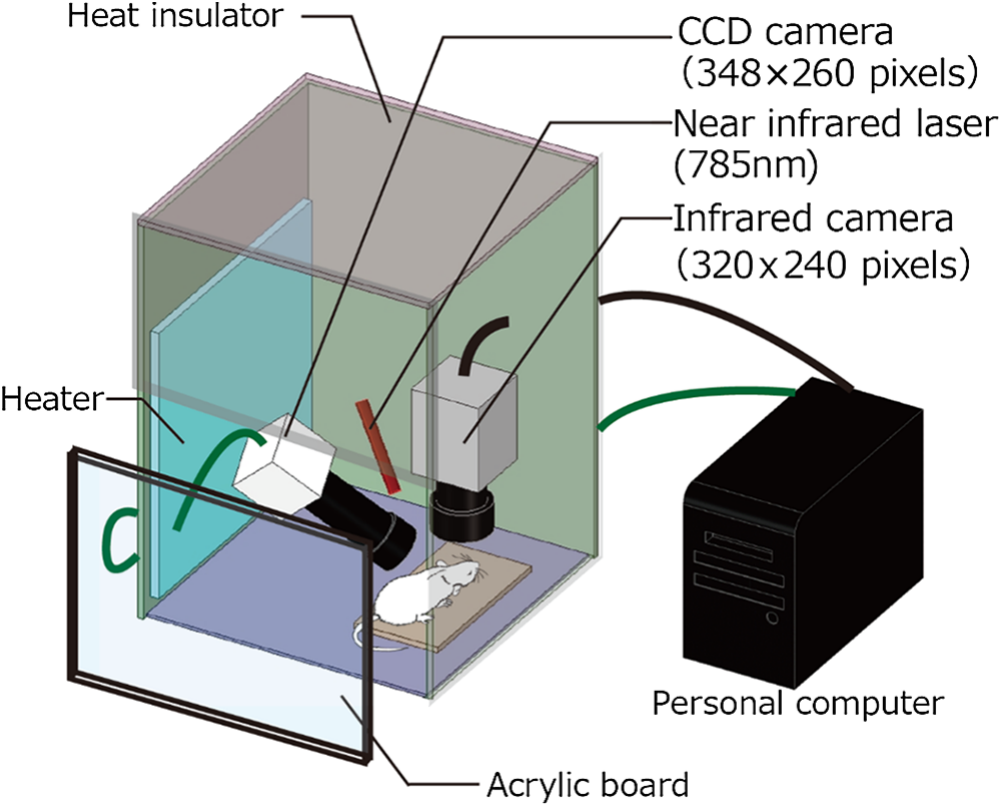
Schematic of the simultaneous imaging system. A near-infrared laser and a CCD camera were used to construct a laser speckle imaging system to evaluate the 2-D distribution of cerebral blood flow changes. An infrared camera measures the temperature distribution of the rat cortex through a cranial window. A computer is controlling and synchronizing image acquisition from the two cameras. The imaging system is covered with 20-mm-thick insulation boards. A part of the cover is a clear acrylic board, which enables observation of the interior. A heater controls and maintains the temperature in the box. A black curtain covers the box to reduce light source noise from outside during measurement.

The animal experiments were performed in accordance with the Guidelines for Animal Experimentation from the Ethics Committee of Kyoto University (Permit Number: Kyo Med 15566 and 16202). All surgeries were performed with the rats under anesthesia, and all efforts were made to minimize suffering. Seven male Wistar rats (9 to 12 weeks old) (Japan SLC, Japan) weighing 200 to 270 g were used. The rats were initially anesthetized with isoflurane (2.0% gas) in air through a gas anesthesia mask during a tracheotomy. After the tracheotomy, mechanical ventilation (Independent Dual Output Small Animal Ventilator; NEMI Scientific) was adjusted to maintain physiological parameters within normal ranges under isoflurane anesthesia through the endotracheal tube (0.5% to 1.5% gas). The left femoral artery was cannulated using polyethylene tubing (SP45; Natsume Seisakusho Co., Ltd., Japan) to connect a pressure transducer (KN-211; Natsume Seisakusho Co., Ltd., Japan) for monitoring the systemic blood pressure. Body temperature was maintained at 36.5°C to 37.0°C using a heating pad, and a rectal thermometer (FST-HPS; Fine Science tools, Canada) was used to provide feedback control. Another type-T thermocouple was also inserted 7-cm deep in the rectum to measure body core temperature (RET-2; Physitemp Instruments Inc.). Mannitol (2  g/kg, i.p.) [D-(-)-Mannitol; nacalai tesque, Japan] was administered to prevent brain edema after removing the skull. The rats were fixed to a stereotaxic holder (SR-5R-HT; NARISHIGE, Japan). The skin on top of the skull was removed to expose the location of the bregma. A 3-×3-mm square cranial window 2- to 5-mm posterior and 2- to 5-mm left lateral to the bregma was drilled in the skull to expose the somatosensory cortex leaving the dura mater intact. During the drilling process, the skull was cooled using a saline solution to prevent heat damage. Cannulation of the caudal vein was performed to administer α-chloralose during measurement. Following these procedures, the stereotaxic holder that contained the anesthetized rats was moved to an imaging system (Fig. 1).

### 2.2 Imaging System

#### 2.2.1 Vessels imaging

The architecture of rat cortical surface blood vessels was imaged using a digital camera (D800E; Nikon, Japan) and a stereoscopic microscope (SMZ645; Nikon, Japan). Following the blood vessels imaging, a few small pieces of aluminum foil cutting the triangle shape were placed on the cortical surface as landmarks for postprocessing matching positions between a blood flow image and a temperature image. The cortical surface including the aluminum foil pieces was captured using this system.

#### 2.2.2 Brain thermal imaging

Figure 1 shows our experimental setup. This imaging system was composed of an infrared (IR) thermal imager and a laser speckle imager. In the IR thermal imaging, the temperature distribution of the cortex was measured using an IR camera (Merlin Mid; FLIR Systems Inc.). This camera contained an indium antimonide IR sensor cooled by a closed-cycle cryocooler. The temperature measurement resolution of the camera was as small as 0.018°C. The camera sensor was an array of 320×256  pixels covering 3.84×3.07  mm of the object with 2.5× objective (322.0005.03 MWIR Microscope 2.5× objective, FLIR Systems Inc.). The IR camera is sensitive to IR light in the 3- to 5-μm bandwidth, using an internal bandpass cold filter that sets the operating spectral range to this bandwidth.

#### 2.2.3 Cerebral blood flow imaging (laser speckle imaging)

The cerebral blood flow changes in the rat somatosensory cortex were measured by laser speckle imaging. Laser speckle imaging has been widely used to image relative changes in cerebral blood flow without contact in animal models *in vivo*. This system simply includes a laser for illuminating the tissue surface and a camera for detecting the back-scattered light. A near-infrared laser diode (wavelength: 785 nm, power output: 4.5 mW, LDU33-785-4.5; SIGMA KOKI Co., Ltd., Japan) illuminated the exposed left somatosensory cortex. The speckle pattern generated by the reflected light was detected using a monochromatic 12-bit charge-coupled device (CCD) camera (TXG14NIR; Baumer, Switzerland) with 348×260  pixels covering 5×5  mm of the object. To acquire blood flow images at high-spatial resolution, we calculated the relative cerebral blood flow changes from the acquired images using a measurement technique of temporal-domain speckle contrast flow based on the theoretical analysis of speckle images presented by Cheng et al.^26^ (the details about calibrations are described in Sec. 2.3).

#### 2.2.4 Multimodal imaging

The filter of the IR camera can cut off the near infrared laser light for the measurement of blood flow. Thus, the near IR laser light does not interfere with IR thermal imaging. This bandpass of the IR camera made it possible to visualize both temperature and blood flow changes simultaneously. Both images were captured synchronously using a computer.

To achieve high accuracy in the brain temperature measurements, we had to reduce momentary temperature changes and temperature fluctuations due to airflow. We covered the imaging system with a box (W: 600×D: 450×H: 700 mm) that was made from 20-mm thick thermal insulation boards (Styrofoam; Dow Chemical Company) (Fig. 1). A part of the box was open to facilitate setting of the rat on the imaging station and to arrange cables for the measurements. This open part was covered with a transparent acrylic plate (600×450×5  mm) that was removable and enabled observation of the condition of the inside after closing. A heat plate (HP-4530; AS ONE, Japan) was put in the box as an inner temperature-controlling device. The inner temperature in the box was defined as the average at the tip of the IR camera lens and near the cranial window of the rat measured using type-K thermocouples (P-00306; AKIZUKI DENSHI, Japan). By covering the box with a black curtain during the experiment, outside light incidence that may cause image noise could be prevented. Unlike the blood flow image, the temperature image has no information about vascular structures. Before the measurements, we acquired images of the cranial window, including the aluminum foil pieces, using the IR camera and the CCD camera for the image registration. On the other band, the aluminum foil can be visualized through both cameras. We obtained an affine matrix based on each of the three coordinates of the aluminum vertex points. After these image acquisitions, the pieces of aluminum foil were removed from the cranial window.

### 2.3 Data Collection and Analysis

IR images were captured via an image capture board (NI PCI 1424; National Instruments) and saved on a computer as binary data using in-house software written using LabVIEW (National Instruments). The CCD images for the laser speckle imaging were transferred to the computer using the Gigabit Ethernet communication protocol. These CCD images were converted to the blood flow images using laser speckle imaging developed by Cheng et al.^26^ In this method, at first, the speckle contrast (C) is calculated as

$$C_{i,j}=\frac{\sigma }{\left\langle I\right\rangle }=\frac{\sqrt{\frac{1}{n+1}\sum_{l=t-n/2}^{t+n/2}I_{i,j,l}^{2}-{\left(\frac{1}{n+1}\sum_{l^{\prime }=t-n/2}^{t+n/2}I_{i,j,l^{\prime }}\right)}^{2}}}{\frac{1}{n+1}\sum_{l=t-n/2}^{t+n/2}I_{i,j,l}},$$ (1)

where σ is the standard deviation, ⟨I⟩ is the mean value of light intensity. $I_{x,y,t}$ is the intensity of pixel (i,j) at time t and n+1 is the number of speckle images over which the contrast is calculated. In our case, 11 images were used to acquire a blood flow image (n=10). Next, each speckle contrast image was converted to a speckle flow index (SFI) map:

$${\mathrm{SFI}}_{i,j}=\frac{1}{2TC_{i,j}^{2}},$$ (2)

where T is the exposure time. In our case, the exposure time is 8 ms. The SFI is proportional to the blood flow. Finally, the blood flow image is created applying 3×3 spatial filter in each SFI map. These images were calculated using another in-house software program written in LabVIEW to evaluate the relative cerebral blood flow changes and were saved on the computer as binary data. Both temperature and blood flow images were captured at 20  frames/s. Arterial blood pressure was amplified by a strain amplifier (6M92; San-ei, Japan) and collected at a rate of 1000  samples/s via a data acquisition module (NI USB-6216 BNC; National Instruments) to another computer. The mean arterial blood pressure was calculated as the average arterial pressure every 1 s. Estimating heart rate and respiratory rate was accomplished by passing the blood pressure signal through a bandpass filter (fifth-order Butterworth digital filter) such that the passband was centered at the heart rate (5 to 8 Hz) and the respiratory rate (0.7 to 1.0 Hz) frequencies. We calculated interval times from peak to peak to determine the heart rate and respiratory rate with the peak detector virtual instruments (VI) function of LabVIEW. The rectal temperature of the rat and ambient temperatures in the box were also collected at a rate of 1  sample/s via a data logger (TC-08; Pico Technology, UK) to the computer. These data acquisitions and analyses were performed with in-house software written in LabVIEW for each.

Changes in brain temperature and cerebral blood flow were calculated using previously described processing methods^6^^,^^27^ implemented in ImageJ (National Institutes of Health). We executed the alignment of the blood flow image (348×260  pixels) to the brain temperature image (320×256  pixels) using an affine transformation to evaluate the same region. The affine transformation was computed using the MATLAB Image Processing Toolbox function, affine2d (Mathworks). This transform is obtained from the relation between three distinct points. To use the affine2d function, the 3×3-affine matrix was defined from each of the three coordinates of the aluminum vertex points. The image transformation was applied to the stack of blood flow images. The temperature images and transformed blood flow images were trimmed using a region of interest of 150×180  pixels. Each pixel represents an area of 12×12  μm in the images.

Temporal analyses of cerebral blood flow and temperature were performed by averaging intensity values within the entire image region of interest (150×180  pixels) on the stack image using the ImageJ, as the image included some misregistration caused by nonlinear deformations such as vessels. In addition, to investigate the relationship between the blood flow and the temperature, we averaged these temporal changes over the entire image region from seven animals.

To visualize the relationship between the cerebral blood flow and the brain temperature, correlation maps between the blood flow and temperature changes were created. Pearson correlation coefficient values were calculated on the basis of one pixel in a time sequence for 5 min. Each pixel (i,j) in a correlation map is represented by a correlation value $r_{ij}$ ($-1\leq r_{ij}\leq 1$). A two-dimensional (2-D) distribution of the correlation values at each pixel depicted the correlation map of the 5 min changes between the blood flow and the temperature. We created the maps in every 5 min. The original images were processed by a denoising filter in both temporal and spatial directions (Gaussian Blur 3D: $\sigma_{i}=2\text{ pixels}$, $\sigma_{j}=2\text{ pixels}$, t=100  frames) using ImageJ before a calculation. The correlation value was calculated using in-house software written in LabVIEW. The relationship between the cerebral blood flow and the brain temperature was visually inspected on representative vessels.

### 2.4 Protocols

**Fig. 2:**
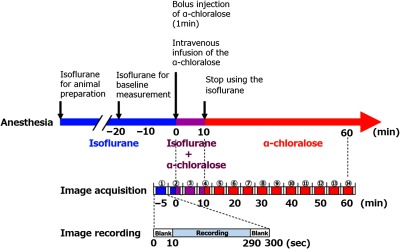
Study protocol. The colored horizontal arrow shows how the anesthesia was administered. The black vertical arrows indicate the timing of each event. The middle crossbar shows the sequence of image actuation. The other crossbar of the bottom indicates a detail time sequence of an image recording trial.

Changes in brain temperature were achieved using two different anesthetics (isoflurane and α-chloralose). The method of brain temperature control was the same as used in Zhu’s study.^25^ The time course of this protocol is shown in Fig. 2. The rat was first anesthetized with 2.0% isoflurane. We waited at least 20 min until the physiological parameters stabilized following the 2.0% isoflurane inhalation. Recording of the brain temperature and cerebral blood flow was started under baseline conditions for 10 min after the steady state (isoflurane only). Following the baseline recording, the anesthesia was added to a 5.0  mL/kg intravenous bolus of α-chloralose and administered over 1 min with isoflurane followed by continuous intravenous infusion at 5.0  mL/kg/hr (isoflurane + α-chloralose). The isoflurane anesthesia was removed 10 min after the α-chloralose infusion (α-chloralose only). We maintained the α-chloralose condition for 50 min. Image actuations were divided into 14 trials. Each trial took 5 min, including 10-s blanks at the beginning and end, which gave a total recording time of 280 s. The missing data during the blanks were interpolated linearly. Therefore, each trial had 5600 temperature images and 5600 blood flow images, respectively. To investigate responses related to the bolus injection (at 0 min in Fig. 2) and elimination of the inhalation anesthesia (at 10 min in Fig. 2), the middle of the second and fourth trials matched the time points when each event occurred. We applied the Gaussian filtering in both the temporal and spatial directions to the temperature and blood flow images for smoothing using ImageJ.

### 2.5 Statistical Analysis

Experimental data are expressed as means±SD, and error bars in graphs indicate SEs. The statistical significance of the changes was assessed by one-way repeated measures (ANOVA) for temporal changes and the post hoc Dunnett’s test (multiple comparisons versus a control). Data were analyzed using R (Version 2.15.2). The level of statistical significance was set at P<0.05.

## 3 Results

### 3.1 Temperature Controls

**Fig. 3:**
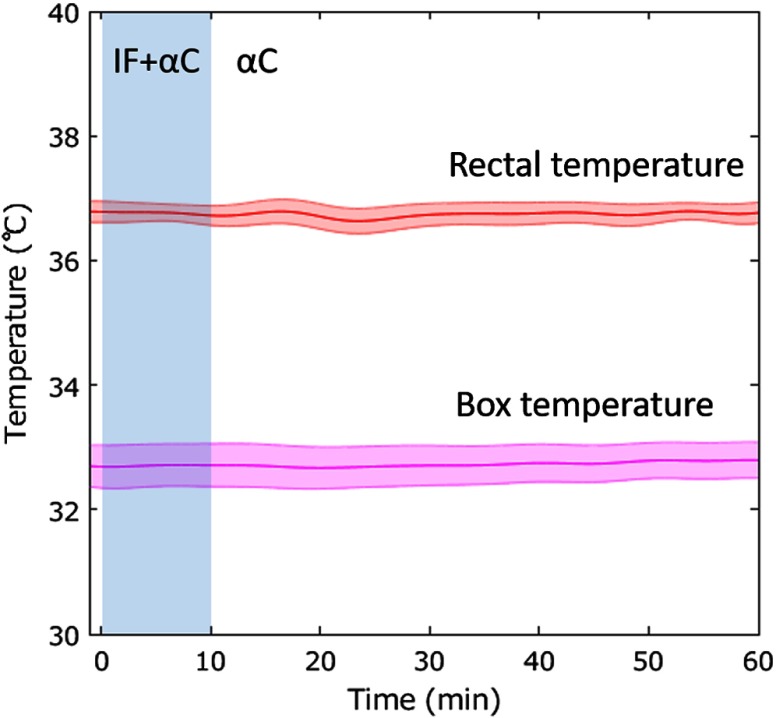
Temporal change in rat rectal temperature averaged from seven animals and ambient temperature in the box averaged from seven trials. Values are means (line) ± SE (a fill pattern with similar color of the line). The shade in the graph indicates overlapping usage of isoflurane (IF) and α-chloralose (αC).

The averaged time courses of the rat rectal temperature as core temperature and the ambient temperature in the box covering the imaging systems during experiments are shown in Fig. 3. These temperatures remained extremely consistent (Rectal: 36.74°C±0.45°C, Box: 32.73°C±1.16°C) throughout the experiments.

### 3.2 Physiological Changes

**Fig. 4:**
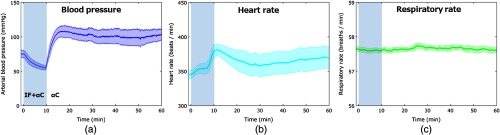
Temporal changes in the rat mean arterial blood pressure, heart rate, and respiratory rate averaged from seven animals. Values are means (line) ± SE (a fill pattern with similar color of the line). The shade in the graph indicates overlapping usage of IF and αC.

The mean arterial blood pressure, the calculated heart rate, and respiratory rate from the femoral artery blood pressure data are shown in Fig. 4. The mean arterial blood pressure [Fig. 4(a)] decreased for 10 min significantly (P=0.027) from the initial control value following infusion of α-chloralose (at 0 min) with isoflurane anesthesia (from 75.21±14.9 to 57.13±9.71  mmHg). However, blood pressure increased immediately after suspension of isoflurane usage (at 10 min) and reached a peak value (107.51±20.6  mmHg) at 16 min. After the peak, the blood pressure remained almost stable. The heart rate calculated from the arterial blood pressure [Fig. 4(b)] increased significantly (P=0.042) for 10 min after the infusion of α-chloralose (from 345.39±18.1 to 378.56±28.8  beats/min) while the blood pressure decreased. Subsequently (10 min after the infusion), the heart rate decreased immediately by about 20  beats/min while the blood pressure increased. From 30 min after the infusion, the heart rate slightly increased. The respiratory rate calculated from the blood pressure data [Fig. 4(c)] remained substantially constant because of the mechanical control.

### 3.3 Blood Flow and Brain Temperature Images

**Fig. 5:**
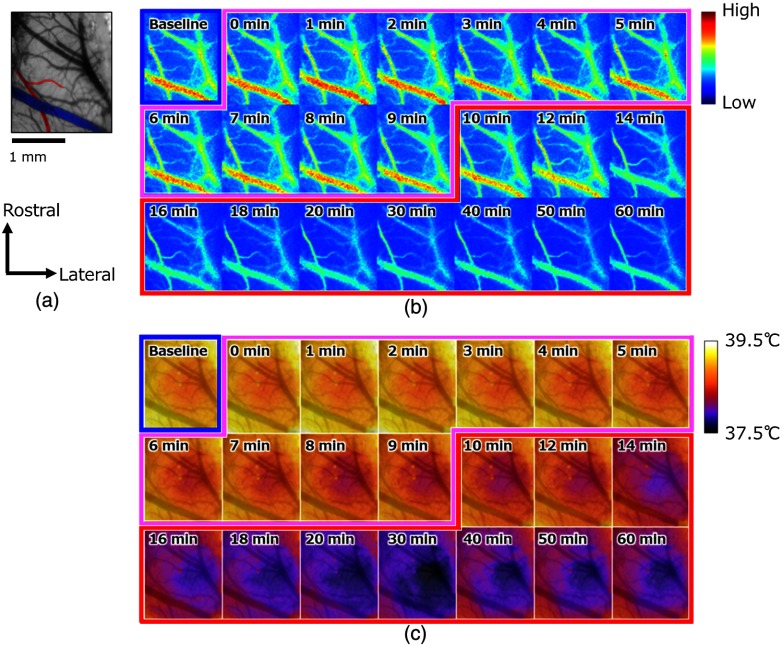
(a) A representative camera image of the cranial window. The image allows a characteristic vein (blue) and an artery (red) to be easily distinguished. (b) Averaged images of speckle flow indices indicate cerebral blood flow changes in the cerebral cortex accompanying the anesthesia for one rat. The figures on each image indicate the elapsed time from the beginning of the α-chloralose injection. (c) Averaged images of temperature in the cerebral cortex accompanying the anaesthesia for one rat. The figures on each image indicate the elapsed time from the beginning of the α-chloralose injection. The different colors of the framework indicate the status of anesthesia: isoflurane (blue), α-chloralose (red), and mix (purple). The camera image taken at baseline is overlaid on each image to add vascular structural information.

**Fig. 6:**
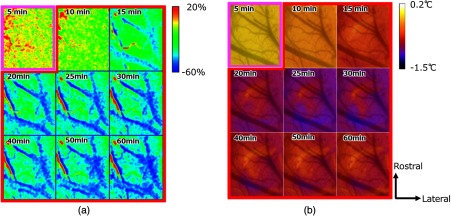
(a) A representative sequence of images showing the percent changes in cerebral blood flow in response to the anesthesia from one rat. (b) sequence of images showing the temperature differences from the baseline image in response to the anesthesia from one rat. The different colors of the framework indicate the status of anesthesia: both isoflurane and α-chloralose (purple) and α-chloralose (red). The camera image taken at baseline is overlaid on the temperature images to add vascular structural information. The figures on each image indicate the elapsed time from the beginning of the α-chloralose injection.

Figure 5(b) shows an example of cerebral blood flow images mapping speckle flow indices that describe the blood flow changes in the cranial window. Figure 5(c) also shows the acquired IR thermal images with mapping of the absolute temperatures in the same region of the same rat cerebral cortex during anesthesia while switching from isoflurane to α-chloralose in the subject. The percent change in the cerebral blood flow obtained by subtracting and dividing the baseline from each image, and the brain temperature change obtained by subtracting the baseline from each image in Fig. 6. We could not observe the vascular structure on the temperature images, although the blood flow images provided clear 2-D structural information about the cortex vasculature. To understand the spatial information based on the vascular structure on the temperature images, the images showing temperature change were overlaid on a digital camera image of the cranial window at a baseline condition [Figs. 5(c) and 6(b)]. The values of the SFI for the large vein indicated in blue in Fig. 5(a) were higher than those for other vessels throughout the experiment. In addition, we observed that the index values in the center of the vein were higher than the values near the vessel wall from baseline to 12 min after the α-chloralose injection. Just after the injection of the α-chloralose [from 0 to 2 min in Fig. 5(b)], the value of the SFI increased transiently in the vessels. After dissipation of this overshoot, under both isoflurane and α-chloralose anesthesia, the blood flow decreased but did not show dramatic changes. During only the α-chloralose anesthesia, the blood flow was significantly decreased, especially in the cerebral vessels. The running of red and blue parts in parallel near the artery resulting from a misregistration appeared in Fig. 6(a) from 15 to 60 min. The artery indicated in red in Fig. 5(a) became thinner and changed shape in Fig. 5(b) accompanied by reduction in the blood flow. In our imaging technique, it is difficult to adjust the misregistration of the artery. As a result, the misalignment of the vessel led to misunderstanding that the percent changes in the blood flow increases that appeared in the artery during blood flow had decreased totally.

The distribution of the brain temperature varied, and the temperature of the center of the cortex was lower than the temperature of the surrounding area at baseline [Fig. 5(c)]. The temperature of the cortex appeared to decrease while maintaining the temperature distribution during both anesthesia usage (from 0 to 9 min), irrespective of the vascular arrangement. During only the α-chloralose anesthesia, the temperature also decreased ∼0.5°C until 30 min. From 40 to 60 min, an ∼0.1°C increase in the brain temperature was observed. The temperature differences from the baseline [5 and 10 min in Fig. 6(b)] indicate a reduction in the temperature. The decreases in brain surface temperature were observed in the center of the region [from Fig. 5(c)]. In fact, however, the temperature decreased not only in the center of the cortex, but also in the surrounding area until 30 min [from Fig. 6(b)]. During the α-chloralose anesthesia, there were small areas in which the temperature differences near the center of the cranial window were smaller than those in the surrounding areas [Fig. 6(b)]. However, the areas with less temperature variation did not match the vascular distribution. Following the temperature reduction until 30 min, the brain temperature increased again. The pial vein region in the rat brain showed a clear temperature decrease relative to the region surrounding the vein from 25 to 60 min.

### 3.4 Relationship between Changes of Blood Flow and Temperature

**Fig. 7:**
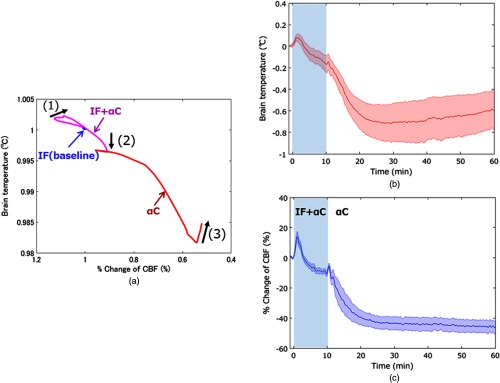
(a) Relationship between temporal change in cerebral blood flow and brain temperature averaged over the entire image region from seven animals. Three black arrows [(1), (2), and (3)] indicate directions of travel and ranges of decoupling between the temperature and the blood flow. (b) Temporal change in temperature averaged over the entire image region from seven animals. (c) Graph illustrating the percent change in cerebral blood flow averaged over the entire image region from seven animals. Values are means (line) ± SE (a fill pattern with similar color of the line). The shade in the graph indicates overlapping usage of IF and αC.

Figure 7(a) shows the interactive relationship between the cerebral blood flow changes and the brain temperature changes over time. Each data point indicates the ratio of the change from the baseline value. In our study, it is difficult to indicate the averaged images from seven animals as vessel structures on the cortical surface are quite different among them. Instead of showing the averaged images, Figs. 7(b) and 7(c) show that temporal changes in the brain temperature and the percent changes in cerebral blood flow averaged over the entire brain region from seven animals, respectively. Both the blood flow and temperature increased transiently from the initial control value (about 13% and 0.08°C increase, respectively) following the α-chloralose infusion. The initial increases reached a peak in 1 min. The mean temperature decreased during the isoflurane and α-chloralose anesthesias after the peak, and the value at 10 min was significantly (P=0.042) lower than the control value. The mean blood flow change also decreased by about 10% between the value at the peak and at 6 min after the α-chloralose injection under the isoflurane and α-chloralose anesthesias. The change in the blood flow remained flat from 6 to 10 min while the temperature continued to decrease. After the isoflurane anesthesia was removed, the blood flow indicated significant exponential (P<0.001) reduction from 10 to 60 min. We also observed similar temperature reduction until ∼30  min. However, following this reduction, the brain temperature started to increase gradually to 60 min. The relationship between the cerebral blood flow and brain temperature was not correlated partly. During the isoflurane and α-chloralose anesthesia, at first, both blood flow and temperature increased. Following these increases, we observed a discrepancy between the decrease in the cerebral blood flow change and the increase in brain temperature change, and the direction of change was inclined upward left [arrow (1) in Fig. 7(a)]. After this decoupling, we confirmed that both the blood flow and temperature were decreasing. Following these decreases, the steady state of the blood flow and the decrease in temperature were appeared, and the direction of change was downward [arrow (2) in Fig. 7(a)]. During the α-chloralose anesthesia, until 20 min after the α-chloralose injection, both the blood flow and the temperature were decreasing. However, from 20 to 60 min after the injection, we also observed another discrepancy between the decrease in cerebral blood flow change and the increase in brain temperature change, and the change direction was also inclined upward left [arrow (3) in Fig. 7(a)].

### 3.5 Correlation of Brain Temperature Changes with Cerebral Blood Flow Changes

**Fig. 8:**
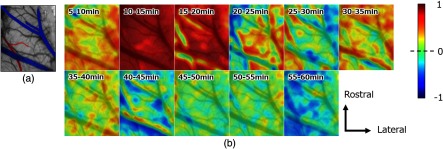
(a) A representative camera image of the cranial window. Colored parts on the representative vessels (red—artery, blue—vein) indicates positions where some characteristic correlations are observed. (b) Images mapping the Pearson correlation coefficients between brain temperature changes and cerebral blood flow changes for every 5 min. The camera image taken at baseline is overlaid on the temperature images to add vascular structural information. The figures on each image indicate the time interval for calculating the Pearson correlation coefficient.

Figure 8 shows maps indicating the spatial distribution in the correlation coefficients between the cerebral blood flow and brain temperature every 5 min. The images were overlaid on a digital camera image of the cranial window at a baseline condition. As a result of a visual inspection for the relationship between the blood flow and the temperature, correlations were observed along the vessels indicated in red and blue in Fig. 8(a). During the mixed anesthesia (5 to 10 min), the correlation coefficients were mostly low because the blood flow was almost constant and the temperature decreased. However, the correlation coefficients for the areas surrounding the vessels tended to be higher than those of the other areas. After removing the isoflurane (10 to 15 min, 15 to 20 min), there were remarkable positive correlations between cerebral blood flow and brain temperature changes, but we found that the correlation coefficients near the vessels became smaller from 15 to 20 min. From 20 to 45 min, there were no strong correlations between the temperature and blood flow. Moreover, the correlation coefficients in the vessels were negative from 20 to 25 and 25 to 30 min. On the other hand, we found positive correlations for the vessels from 30 to 35, 35 to 40, and 40 to 45 min. From 45 to 50 and 50 to 55 min, only weak negative correlations were observed. We observed that there were remarkable negative correlations overall from 55 to 60 min. These negative correlations indicated decoupling between the changes in brain temperature and cerebral blood flow.

## 4 Discussion

In this study, we demonstrated an effective method for simultaneous imaging of cerebral blood flow and brain temperature by combining laser speckle imaging and brain thermal imaging. This technique can be helpful for understanding neurovascular metabolic coupling, quantitative functional changes in the brain, and for monitoring some cerebral disorders. In addition, our development system uses only one laser light and needs no complicated mechanical and optical systems. Furthermore, temperature measurement with high resolution to evaluate metabolic changes can be used without calibration and must be more quantitative than the phosphorescence method. The imaging can only evaluate the cortical surface, but the 2-D distribution of each change can achieve high spatial–temporal resolution. Using this imaging system, we successfully showed a spatiotemporal discrepancy between the cerebral blood flow and brain temperature.

Absolute brain temperature changes are some of the most critical parameters that indicate metabolic changes in the brain. Brain thermal imaging has shown that local brain temperatures increase in rat somatosensory cortex with whisker stimulus.^6^ This local brain temperature change is caused not only by metabolic changes but also by cerebral blood flow changes regarding heat transport. Laser speckle imaging was combined with brain thermal imaging to enable evaluation of blood flow changes in conjunction with brain temperature changes.

The imaging system shown in Fig. 1 uses two imaging cameras to enable simultaneous imaging. The imaging works without optical interference because these cameras detect different wavelengths: 3 to 5  μm in the thermal camera and 0.785  μm (785 nm) in the other CCD for blood flow imaging. The image shift caused by the two cameras was corrected by an image-processing technique called affine transformation. By covering the imaging system with a box of insulation and a black curtain, we successfully reduced temperature and light noise and achieved stable measurements. However, the 60-min thermal imaging could increase the shot noise of the camera although it was cooled by a closed-cycle cryocooler.

We have successfully tested the ability of our system to measure the rat cortical surface during two kinds of anesthesia: isoflurane and α-chloralose. Both isoflurane^28^ and α-chloralose^29^ induce hypothermia that can lead to death in severe cases. Preventing anesthesia-induced hypothermia enabled maintenance of the rat rectal temperature as body core temperature at an almost constant value (36.5°C) using a heating pad (Fig. 1). Moreover, we also controlled the ambient temperature around the rat using an insulated box and a heater. In the rat, the brain temperature is affected by not only the cerebral blood flow,^30^ but also by the temperature difference between the brain surface and ambient air.^31^ Especially, because the cortical surface of a rat is exposed through the cranial window, as in our experiments, the ambient temperature becomes important. Reducing the temperature difference between the brain surface and ambient air, the temperature in the insulation box was set higher (33°C) than that of the experimental room (25°C). As a result, we observed decoupling between the body core temperature and brain temperature. This finding indicated that we succeeded in controlling the brain temperature only using anesthesia while maintaining near-constant body temperature.

Isoflurane and α-chloralose are also known to strongly effect cerebral blood flow and metabolism. Cerebral metabolism is reduced by isoflurane^32^ and α-chloralose.^33^ Alpha-chloralose also causes a significant decrease in cerebral blood flow.^24^ On the other hand, isoflurane increases cerebral blood flow after administration because of its vasodilation effect.^21^^,^^22^ During the mixed anesthesia period using both isoflurane and α-chloralose, we found a decorrelation in which blood flow remained constant, but brain temperature decreased (Figs. 7 and 8). This discrepancy between these changes could have been caused by differences in the anesthetic effects on blood flow and metabolism. Because isoflurane had already been used for sedation during animal preparation before the measurement, the baseline cerebral blood flow must have been higher than that under the usual arousal condition without isoflurane.^34^ The counteraction of these anesthetics caused a plateau stage in the blood flow changes during the mixed anesthesia period [Fig. 7(b)]. Although there were no strong correlations on the correlation map from 5 to 10 min between the temperature and blood flow, the correlation coefficients in the large vessels tended to be larger than those in the other regions [Fig. 8(b)]. The brain temperature decreases were uniform in the cranial window. In contrast, the blood flow changes showed a special distribution during this period (Figs. 5 and 6). Tsurugizawa et al.^35^ reported that blood oxygen-level-dependent signal increases in tissue regions <2.0% and <2.5% isoflurane were different from those in large vessels in rats. Masamoto et al.^34^ measured vessel diameters and red blood cell speed on the cortical surface of rats anesthetized with either isoflurane or α-chloralose by multiphoton excitation fluorescence microscopy. Consequently, they found that the effect of anesthesia on capillary diameter was opposite to the arterial response, and the speed of red blood cells was lower under α-chloralose relative to that under the isoflurane condition. These differences between the isoflurane and α-chloralose effects on the cerebral cortex caused a distribution in the correlation coefficient [Fig. 8(b), 5 to 10 min].

Alpha-chloralose reduces both cerebral blood flow and metabolism, as stated above. In addition, the elimination of vasodilation due to suspending the use of isoflurane would cause further blood flow reduction. Hence, after stopping the isoflurane, cerebral blood flow and brain temperature would decrease for about 20 min. Moreover, we observed extremely strong correlations between the changes in temperature and blood flow from 10 to 15 min and from 15 to 20 min [Fig. 8(b)]. In the correlation map from 15 to 20 min, however, there were uncorrelated parts in the vessels. Then, weak negative correlations appeared in the vessels [20 to 25 min in Fig. 8(b)] and spread from the large vessels throughout the whole region [25 to 30 min in Fig. 8(b)]. These negative correlations resulted from the blood flow decrease and temperature increase. The relative decrease in blood flow was ∼40% in the vessels up to this point. The brain temperature is determined by the balance between heat caused by metabolic change and the heat dissipation caused by the cerebral blood flow.^36^ From 30 to 40 min, the blood flow decreases leveled off, and the rates of change in the temperature and blood flow were small. Therefore, positive correlations appeared once again [30 to 35 and 35 to 40 min in Fig. 8(b)]. Subsequently, the negative correlations spread throughout the overall cortex [45 to 50, 50 to 55, and 55 to 60 min in Fig. 8(b)]. In this phase, the blood flow was slightly decreasing. On the other hand, the brain temperature was significantly increasing, although the brain cerebral metabolism was also reduced by the effect of α-chloralose. The cerebral blood flow may have been close to its lower limit and unable to function as a brain coolant at that point. The overwhelming cooling effect of increased blood flow versus the marginal brain heating from increased metabolic rate has been reported.^37^ It is also known that brain temperature is elevated in acutely ischemic human brain.^38^ The discrepancy shown by the negative correlations may have been caused by a failure in thermoregulation in the brain.

To evaluate our imaging system, we caused changes in brain temperature and cerebral blood flow using isoflurane and α-chloralose in a manner similar to that described by Zhu et al.^25^ These anesthetics, which are widely used in rodents for physiological studies and functional imaging, had effects not only on the brain, but also on the systemic circulation of the rats (Fig. 4). Following the bolus infusion of α-chloralose, transient increases in cerebral blood flow and brain temperature were observed for 1 min. Bolus injections of magnetic resonance agents into the tail vein of rats have been shown to cause slight alterations in cerebral blood flow.^39^ Moreover, the blood flow elevation may indicate the response to tactile stimulation of the tail over the highly elaborated somatic sensory cortex accompanying the injection. In fact, we created the cranial window above the somatosensory cortex on the sensory map of the rats,^40^^,^^41^ including the sensory area corresponding to the trunk and tail stimulation. Tail pinching of rats has also been shown to elicit brain temperature changes.^42^ The vascular distribution of the artery in the cranial window was almost straight during the isoflurane anesthesia (∼10  min). However, after only using α-chloralose, we observed the running of red and blue parts in parallel near the artery appeared in Fig. 6(a) from 15 to 60 min. These parallel parts would be derived from deformations of the artery. This deformation of the blood vessel caused an image registration error. The gap in blood vessel structure produced incorrect results in the subtraction images, such as blood flow increases [Fig. 6(a)]. Thus, to prevent this kind of error and the resulting misunderstanding, careful validation of spatiotemporal image differences is needed.

These results indicate that changes in brain temperature related to cerebral metabolic changes did not always correspond to spatial and temporal cerebral blood flow changes. Therefore, this technique could potentially be used to detect areas of activation and disorder and to evaluate their strength by measuring the absolute change in temperature and relative change in blood flow.

We developed a multimodal imaging system capable of simultaneous brain temperature and cerebral blood flow measurements on the cortical surface of rats. We also demonstrated the performance of this optical imaging system by observing the brain surfaces of rats while using two anesthetics (isoflurane and α-chloralose) to control cerebral blood flow and metabolism. The simultaneous observations of temperature and blood flow should lead to a greater understanding of neurovascular metabolic coupling by separating the effects of metabolism and blood flow. Additionally, a more detailed understanding of brain temperature and blood flow changes could lead to better diagnosis of cerebrovascular diseases. Simultaneous temperature and blood flow imaging can be a useful tool for obtaining a 2-D distribution of neural activities and illuminate details of neurovascular metabolic coupling and cerebrovascular diseases.
